# Inferring transcriptomic cell states and transitions only from time series transcriptome data

**DOI:** 10.1038/s41598-021-91752-9

**Published:** 2021-06-15

**Authors:** Kyuri Jo, Inyoung Sung, Dohoon Lee, Hyuksoon Jang, Sun Kim

**Affiliations:** 1 Department of Computer Engineering, Chungbuk National University, Cheongju, 28644 Korea; 2 Interdisciplinary Program in Bioinformatics, Seoul National University, Seoul, 08826 Korea; 3 Department of Computer Science and Engineering, Seoul National University, Seoul, 08826 Korea; 4 Institute of Engineering Research, Seoul National University, Seoul, 08826 Korea; 5 Bioinformatics Institute, Seoul National University, Seoul, 08826 Korea

**Keywords:** Bioinformatics, Gene expression analysis

## Abstract

Cellular stages of biological processes have been characterized using fluorescence-activated cell sorting and genetic perturbations, charting a limited landscape of cellular states. Time series transcriptome data can help define new cellular states at the molecular level since the analysis of transcriptional changes can provide information on cell states and transitions. However, existing methods for inferring cell states from transcriptome data use additional information such as prior knowledge on cell types or cell-type-specific markers to reduce the complexity of data. In this study, we present a novel time series clustering framework to infer TRAnscriptomic Cellular States (TRACS) only from time series transcriptome data by integrating Gaussian process regression, shape-based distance, and ranked pairs algorithm in a single computational framework. TRACS determines patterns that correspond to hidden cellular states by clustering gene expression data. TRACS was used to analyse single-cell and bulk RNA sequencing data and successfully generated cluster networks that reflected the characteristics of key stages of biological processes. Thus, TRACS has a potential to help reveal unknown cellular states and transitions at the molecular level using only time series transcriptome data. TRACS is implemented in Python and available at http://github.com/BML-cbnu/TRACS/.

## Introduction

The emergence of high-throughput technologies enabled a large number of cellular parameters to define a cell state, including mRNA, histone modifications, DNA modifications and cell surface proteins^1^. In particular, transcriptome data contain cell-specific information. In humans and other organisms, nearly every cell contains the same genes, but different cells show different patterns of gene expression. These differences are responsible for the many different properties and behaviours of various cells and tissues, both in health and disease^2^. Since cells make transitions over time, time series transcriptome data can be useful for predicting transitions of cell states as well. Liu et al. used Global nuclear Run-On sequencing (GRO-seq), RNA sequencing (RNA-seq), and histone-modification Chromatin ImmunoPrecipitation sequencing (ChIP-seq) to reveal lag between transcription and steady-state RNA expression and to identify dynamic transcriptional signatures across the cell cycle such as a large amount of active transcription during early mitosis^3^. van Galen et al. used single-cell transcriptome data and genetic mutation information for relationship among cell types to analyse acute myeloid leukemia (AML) heterogeneity that resides within a complex microenvironment that complicates efforts to understand contribution of different cell types to disease progression^4^. Leveraging the power of single-cell data and cell type information, Grün et al. developed VarID, a computational method that identifies locally homogenous neighbourhoods in cell state space and reveals pseudo-temporal dynamics of gene expression variability^5^. Although these studies are successful in inferring cell states using bulk RNA-seq or single-cell RNA sequencing (scRNA-seq) data, information in addition to transcriptome data is required. In the next section, we discuss how much information transcriptome data can provide for inferring cell states and transitions.

*Feasibility of defining cell states* Recent studies showed that sophisticated computational analysis can infer states of cells and their transitions from transcriptome data. Analysis of scRNA-seq data typically arranges cells in ‘pseudotime’ by their gene expression profiles to add the time dimension to the RNA-seq data for tracking a trajectory of biological transition, which suggests that it is possible to define transitions of cell states from transcriptomes when the time domain is defined^6,7^. In general, clustering of cells is incorporated as an initial step to guide trajectory inference for scRNA-seq data^8^. However, prior knowledge is required to predict the order of the clusters and to assign the cell type to each cluster. For example, a study on acute myeloid leukemia (AML) performed trajectory analysis on scRNA-seq data and re-clustered the results to classify the cells as 15 cell types using the pre-defined cell-type-specific genes^4^. Some of the algorithms for analysing scRNA-seq data try to decode directionality of trajectories as well^8^. The algorithms, however, are designed to order the cells in differentiation such as stem cells, which limits the range of applicable data^9–11^. In addition, clustering cells do not identify the features (genes) that contribute to define cell states.

On the other hand, defining cell states and transition is possible using bulk RNA-seq data, given that the data are measured in specific conditions with uniform cell populations such as a cyclic process where cells are synchronized and a developmental process or perturbation response with a common starting point^12^. When time series transcriptome data is used, grouped genes and their activated time points represent key stages of biological process such as cell cycle phases^3^ or cell types during cell differentiation^4^. One of the widely used methods to detect the gene sets characterizing cell states is clustering gene expression patterns. Chang et al. performed hierarchical clustering on time series mRNA profile in adenocarcinomic human alveolar basal epithelial cells and detected three stages in epithelial-mesenchymal transition (EMT) from the clustering result visualized as heatmap^13^. Clustering genes from gene expression data enables identifying marker genes for each cell state but the requirement of prior knowledge such as the number of cell states can significantly affect the accuracy of clustering.

*Research question* We assume that time series transcriptome data itself has information sufficient enough to predict cell states and their transitions, as described in the previous section. With the availability of a novel framework that uses state-of-the-art computational methods for analysing time series transcriptome data, can we predict both cell states and their transitions without using additional information? In this study, we demonstrate that our proposed computational framework with state-of-the-art clustering algorithms can determine cell states and transitions from bulk-cell or single-cell time series transcriptome data without using any additional information. In the following section, we review clustering algorithms that can be used to analyse time series transcriptome data.

### Related computational methods and limitations

Clustering algorithms on gene expression data can detect distinctive expression patterns of genes, and most of the algorithms aim to improve accuracy by considering time-to-time dependency in expression values. Traditional clustering methods, such as K-means and hierarchical clustering, have been used for many applications and showed high performance in a general clustering problem. However, these methods treat time series observations with *N* time points as *N* dimensional vectors, with an assumption that the time points are evenly sampled and independent. STEM^14^ is a tool for clustering short time series gene expression data. STEM predefines $3^{N-1}-1$ gene expression profiles or clusters from *N* time points assuming that expression values increase/decrease or remain consistent from the previous time point. Due to the large number of predefined profiles, STEM is limited to short time series with 8 time points or fewer. K-Shape^15^ is an adapted clustering algorithm for time series based on the K-means algorithm. It uses a new distance measure called shape-based distance (SBD), clustering similar shapes shifted along the time axis. K-Shape is useful in datasets where similar patterns with different starting points need to be clustered together (e.g., sound waves of multiple species of birds that are all singing at different time points) but is not appropriate when the patterns of different clusters themselves are similar (e.g., expression patterns of cell cycle genes from different phases). Similar to K-means, the number of clusters should be given to K-Shape by the user.

Some of the clustering algorithms assume that the observed gene expression values are inherently generated by an underlying model such as a Gaussian process (GP) and state-space model. GP is a stochastic process with a collection of random variables indexed by time or space where the finite collection of the variables follow a multivariate normal distribution. GP regression incorporates time point information, which can be an informative source of time dependency and noise correction, especially for datasets with uneven sampling rates such as developmental processes and perturbation responses. Bayesian hierarchical clustering (BHC) algorithm^16^ uses GP models and their posterior probabilities for the agglomerative hierarchical clustering with an assumption that gene expression values in a same cluster are generated by one GP. BHC automatically detects the optimal number of clusters by Bayesian model selection. However, BHC is suitable for detecting small groups of genes, not a general landscape of cellular states, as it tends to generate a relatively large number of gene clusters due to its bottom-up strategy. GPclust^17^ introduces a three-level hierarchical structure (cluster-gene-replicate) of the GP models and each GP of clusters is generated by a Dirichlet process (DP). DPGP^18^ uses DP and GP models as well but the number of clusters is automatically set by a model similar to the Chinese restaurant process with a hyperparameter $\alpha$ that determines how likely it is that a new cluster is chosen at a given iteration of the Chinese restaurant process.

ClusterNet^19^ introduces a state-space model to clustering, assuming the observed expression values are generated by hidden state variables. *K*-dimensional hidden variables that represent *K* clusters depend linearly on the previous values of all *K* variables, which helps to infer regulatory relationships ‘between’ clusters unlike the other algorithms. ClusterNet can yield a number of false positive edges due to the limitation of using only gene expression values for network inference. Nevertheless, ClusterNet suggests that the order and relationship between clusters can provide new knowledge.

### Our contributions: a novel computational framework

Although the existing clustering methods try to detect distinctive expression patterns from time series data, the methods are not designed for inferring cell states. To infer cell states and transitions from bulk transcriptome data, clustering algorithm should consider long time series data including unobserved time points and data with temporal dependency. The predicted number of clusters is expected to reflect the number of cell states. In addition, when inferring relationship between clusters, the functional analysis such as enrichment tests needs to be incorporated to filter out false positive relationships derived from expression patterns. Thus, we propose a novel time series clustering algorithm that infer TRAnscriptomic Cellular States (TRACS) with the following features :

- *Selecting the number of clusters* TRACS automatically determines the optimal number of clusters with adapted gap statistics that leverage time point information and consider time-to-time dependency (Gaussian process)
- *Clustering time series* By incorporating adapted gap statistics into clustering, TRACS predicts gene clusters based on temporal patterns of genes adjusted by Gaussian process regression. We will show that the predicted gene clusters correspond to cell states in our experiments.
- *Analysis of the clustering result* TRACS infers the transition of cell states as a cluster network, predicting the order of clusters by their pattern similarity (Shape-Based Distance and Ranked Pairs algorithm) and reducing false positive edges by functional similarity (two-group enrichment test)

## Results and discussion

**Figure 1:**
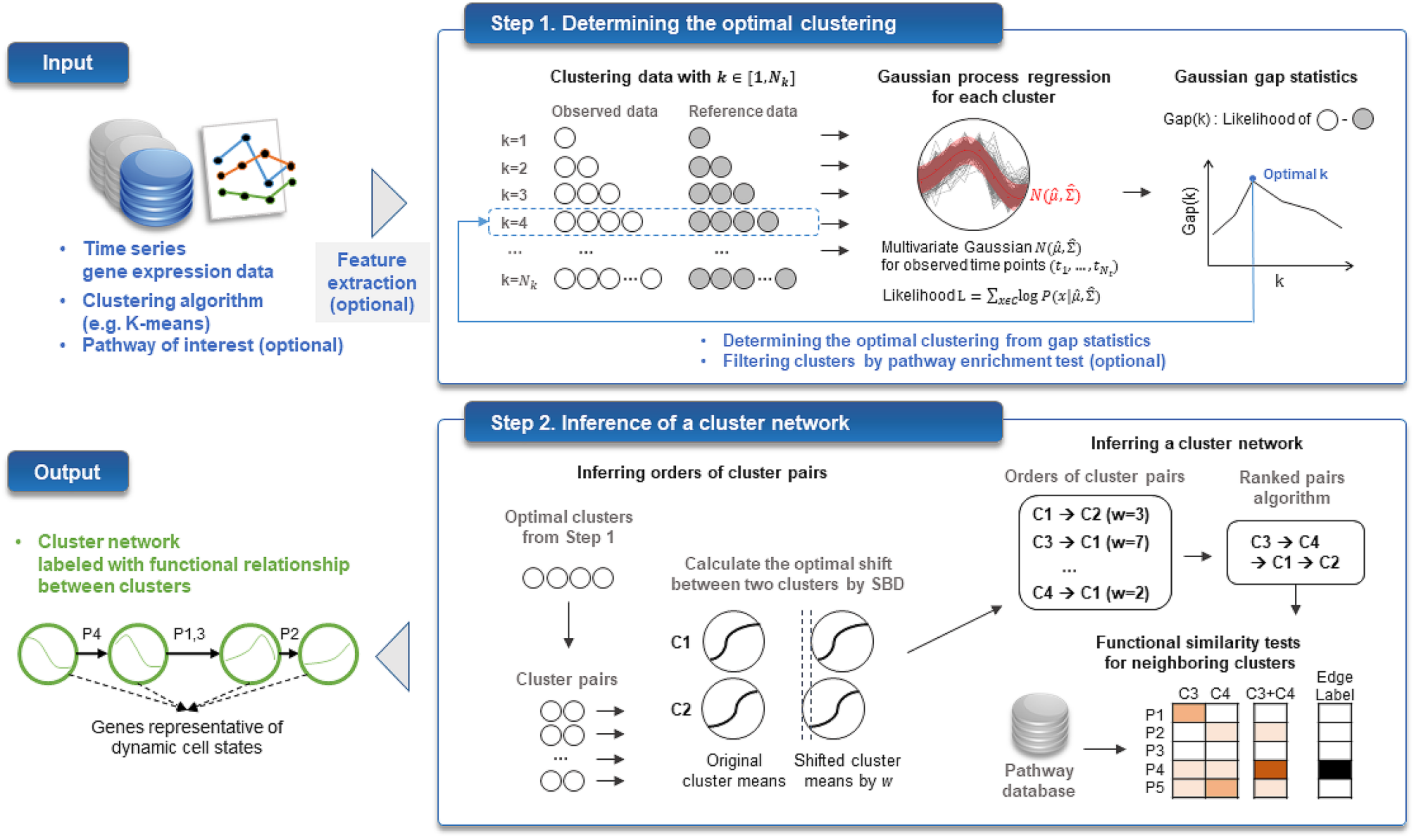
Overview of TRACS. From time series gene expression data, TRACS determines the optimal clustering of time series gene expression data using *Gaussian gap statistics* (Step 1). With the optimal clustering result, a network of clusters is generated and visualized to track the dynamic expression patterns of clusters and their relationships (Step 2). The final output of TRACS is a cluster network describing dynamic cell states and transitions by ordered clusters, where cluster genes imply representative genes of each cell state. Functional relationships between clusters (cell states) are statistically tested and annotated on network edges.

An overview of the proposed framework is illustrated in Fig. 1. TRACS determines the optimal clustering of time series gene expression data using *Gaussian gap statistics* (Step 1). Time series gene expression data are first clustered by a user-selected algorithm such as K-means. Assuming that the gene expressions in *k* clusters are generated from *k* Gaussian processes, Gaussian gap statistics calculate a gap between the likelihood of *k* Gaussian processes from the observed data and from the reference data that are randomly generated with no significant cluster formation. The optimal number of clusters $k_{opt}$ is determined by the gap calculated for a given range of *k*. During Gaussian process regression for each cluster, time point information of gene expression data is used to infer Gaussian means and variances at given time points considering time-to-time dependency. If a pathway of interest is given by the user, TRACS filters out clusters that are not enriched with pathway genes by statistical tests. With the clustering result from Step 1, a network of clusters is generated and visualized to track the dynamic expression patterns of clusters and their relationships (Step 2). For each pair of clusters, shape-based distance (SBD) is calculated, and a shift between two clusters that minimizes SBD is identified. The sign (+/-) of the optimal shift determines the order of two clusters, and all pairwise orders are combined to obtain the overall order of clusters. Functional similarity between neighbouring clusters is tested by a two-group enrichment test to identify a shared function activated over time.

### Descriptions of datasets

The performance of TRACS was evaluated with three sets of gene expression data from scRNA-seq and bulk RNA-seq. We used scRNA-seq data from the research on AML^4^. ScRNA-seq was carried out using Seq-Well protocol to acquire transcriptional data from bone marrow (BM) aspirates. 6915 cells from healthy donors characterized the baseline cellular diversity in BM and the authors distinguished 15 different hematopoietic cell types. The putative differentiation trajectories were inferred by the gene expression similarities of cells, including a continuum of cells from hematopoietic stem cells (HSCs) to monocytes. The authors defined three successive stages of normal hematopoietic development as HSC/Prog, GMP and differentiated myeloid that correspond to five cell types (HSC, progenitor (Prog), granulocyte-macrophage progenitor (GMP), promonocyte (ProMono), monocyte (Mono)) as they show clearly distinguished expression patterns. To generate a time series gene expression data from scRNA-seq data, 2317 cells from the five cell types were sampled and the average gene expression values of each cell type were calculated leading to 5 time points in the order of HSC-Prog-GMP-ProMono-Mono. Based on the process in the original paper, the most variably expressed genes were determined and 21 cell-type-specific genes were added. Removing the genes with zero expression values in more than four time points, the final gene expression data consisted of 360 genes. The original dataset were retrieved from Gene Expression Omnibus (GEO) database (GSE116256).

Two sets of bulk RNA-seq time series data represent a cyclic process and a developmental process, respectively. Cho et al. measured genome-wide mRNA transcript levels during the cell cycle of the budding yeast Saccharomyces cerevisiae^20^. Cdc28-13 cells were collected at 17 time points taken at 10 min intervals, covering nearly two cell cycles. Gene expression data of 6149 genes were downloaded from the Saccharomyces Genome Database (SGD)^21^, among which 220 genes were characterized for each cell cycle phase (early G1, late G1, S, G2, M phase) by the authors according to their transcript levels and biological functions. The dataset from this study has been used as a benchmark for clustering time series as there are few datasets with cluster labels for time series. Chang et al. observed time series mRNA profile in A549 cells (adenocarcinomic human alveolar basal epithelial cells) from TGF-$\beta$-induced EMT samples during 0, 6, 12, 24, 36, 48, 72 and 96 hours. EMT is a metastable process that enables polarized epithelial cells lose their epithelial cell characteristics and acquire a mesenchymal phenotype. The pleiotropic cytokine TGF-$\beta$ is one of the environmental cues and signals that can initiate the EMT process in epithelial cells during wound healing or tumor invasive migration, resulting in the delocalization and/or dissolution of cell-cell junctions and a loss of epithelial integrity^22^. The study reported a three-state model including the partial-EMT state between epithelial and mesenchymal transition and 1,632 genes corresponding to the three states^13^. Gene expression data were downloaded from Gene Expression Omnibus (GEO) (GSE69667). For all datasets, expression values were log- and z-normalized before clustering.

**Figure 2:**
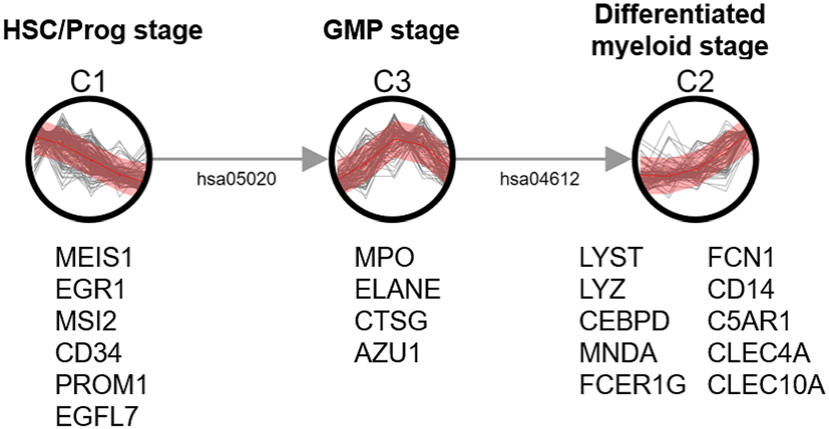
Cluster network of normal BM cells inferred by TRACS. 20 cell-type-specific genes that represent successive stages of normal hematopoietic development (HSC/Prog, GMP and differentiated myeloid stage (ProMono, Mono)) are shown below the assigned cluster. Here and in all following figures, each cluster is represented with a Gaussian process mean (solid red line) and variance (red area, 95% confidence interval) and edges between clusters are annotated with shared biological pathways.

### Cluster networks inferred from time series transcriptome data

#### Single-cell RNA-seq dataset

Figure 2 shows the cluster network inferred by TRACS from the scRNA-seq data. TRACS detected three stages of hematopoietic development (HSC/Prog, GMP and differentiated myeloid stage) in the correct order. The result shows that TRACS can group cell types with similar gene expression patterns and detect distinct cell states regardless of the number of time points (cell types) in the data. According to the original paper^4^, the three stages of hematopoietic development detected by TRACS were the main signature that differentiate normal cells of healthy donors from malignant cells of AML patients. 20 out of 21 cell-type-specific genes clustered correctly to their stages are shown below the network. In particular, CD34 is a predominant marker of HSC and hematopoietic progenitor cells that can represent the HSC/Prog state and CD14 is a typical blood monocyte markers expressed on cells of the myelomonocyte lineage^23,24^. To demonstrate the stability of the proposed method, an additional gene expression data was generated with the increased number of time points by dividing cells from three cell types (HSC, Prog, ProMono) with large populations. In the experiment on the extended data with eight time points, TRACS generated three clusters that represent three stages of hematopoietic development (Supplementary Fig. S1). In addition, TRACS detected *Antigen processing and presentation* (hsa04612) pathway between GMP and differentiated myeloid stages. Antigen processing capacity is known to be induced through differentiation of BM cells starting from GMP to monocytes and monocyte-derived dendritic cells, which is consistent with the TRACS result^25^.

#### Cell cycle dataset

**Figure 3:**
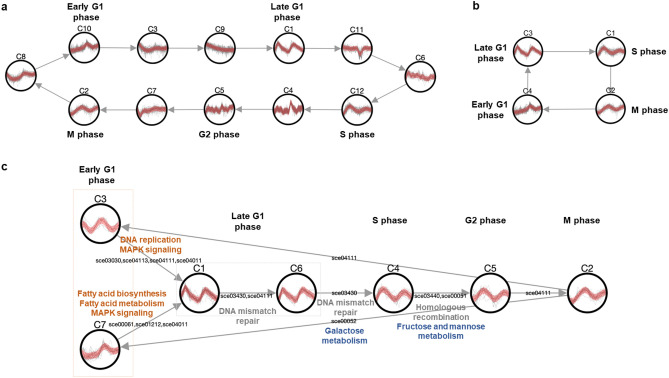
Cluster network of yeast cell cycle dataset by TRACS using (**a**) DEGs (*P*-value > 0.05), (**b**) remaining DEGs after filtering clusters enriched with *cell cycle pathway (sce04111)* from the result of (**a**), and (**c**) 220 labelled genes. In (**c**), repetitive pathway (cell cycle pathway, sce04111) is omitted.

A cluster network is generated by TRACS as shown in Fig. 3. The K-means algorithm is used as a clustering method to find the optimal clustering with Gaussian gap statistics. According to the reference paper^20^, 31, 81, 44, 31 and 34 genes belong to early G1, late G1, S, G2 and M phases, respectively. As the cell cycle dataset contains only 220 genes with labels, which is a relatively small number compared to the total number of genes in yeast, two experiments were designed to evaluate the performance of TRACS using DEGs and 220 labelled genes. The number of genes and the number of clusters used in the two experiments are summarized in Supplementary Table S1. In the first experiment, DEGs are estimated from the gene expression values of the cell cycle dataset and used as input to the TRACS algorithm to show that TRACS can produce informative clusters from larger gene sets that are not limited to functionally labelled genes. As shown in Fig. 3a, TRACS predicted 12 clusters among which 5 clusters were matched 5 cell cycle phases (bold text above circles). The order of clusters predicted by TRACS is still consistent with the order of cell cycle phases. The completeness score^26^ is calculated to check DEGs with the same predefined label (5 cell cycle phases) that are clustered together (Supplementary Table S1). Analysis with DEGs does not degrade the clustering quality of cell cycle genes but rather increases the completeness score since DEGs that are not labelled as cell cycle genes have notably different expression patterns from cell cycle DEGs. As DEGs include genes that are not relevant to cell cycle mechanism, we performed an additional experiment with a context-specific filtering option in TRACS specifying a relevant KEGG pathway as *cell cycle* (sce04111) and a threshold as 0.1. As shown in Fig. 3b, 4 out of 11 clusters that contain cell cycle genes remained after filtering and the order of the clusters were predicted correctly as well.

In the second experiment, 220 genes that are functionally characterized for each cell cycle phase were used to generate a cluster network (Fig. 3c). Each cluster was assigned to a single-cell cycle phase considering which cell cycle phase the largest portion of cluster genes belongs to. Some of the clusters were assigned to the same phase, as the clusters show different expression patterns and biological functions. For example, both cluster 3 and 7 are assigned to the early G1 phase but enriched in different pathways such as DNA replication and fatty acid metabolism, respectively.

TRACS predicted pathways that are activated in two neighbouring cell cycle phases, DNA replication (early G1-late G1), DNA mismatch repair (late G1-S-G2), fatty acid biosynthesis (early G1-late G1) and carbohydrate metabolism (S-G2 and M-early G1). It is known that the formation of the pre-replication complex (pre-RC) occurs during G1 phase and is required for the appropriate initiation of DNA replication in the subsequent S phase^27^. TRACS identified cell division cycle 6 (CDC6) and every component of the MCM2-7 replicative helicase complex except MCM6. The MCM2-7 complex is known as a component of pre-RC that is loaded onto DNA by CDC6 in G1 phase and activated for DNA unwinding^28^. The result implies that TRACS was able to capture the pre-replicative state of cells in G1 phase. The DNA replication checkpoint prevents the accumulation of DNA damage, such as replication blocks or damaged DNA templates, and the checkpoint signal in turn promotes G1-S phase transcription^29^. Checking DNA damage from replication is continued through the intra-S-phase checkpoint^30^ and G2 checkpoint^31^, which corresponds to the functional relationship between clusters suggested from the network. Lipid biosynthesis is also known to coordinate with cell cycle. Inhibition of fatty acid synthesis induces a cell cycle delay at early G1 phase, and a commitment point monitoring the synthesis of lipids is expected to be at the late G1 phase^32^, which explains why pathways related to fatty acid biosynthesis are enriched in early G1-late G1 clusters. Finally, sugar is the most important nutrient for yeast, and the storage of carbohydrates is under cell-cycle control. Storage carbohydrates rise to high levels in the early G1 phase and decrease in late G1 by liquidation to glucose, slowly growing again after S phase^33^.

#### Epithelial-to-mesenchymal transition dataset

**Figure 4:**
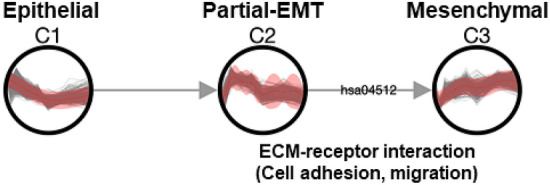
Cluster network of human EMT dataset inferred by TRACS.

TRACS generated a cluster network for the EMT dataset (K-means, $k_{opt}$=3), as shown in Fig. 4. The network is sparsely connected by functional similarity, with no label between the epithelial and partial-EMT clusters. This result is not unexpected because each state of EMT is rather distinct in that state-specific transcription factors cooperatively regulate the transcriptomic dynamics^13^. Additionally, the result is consistent with the GO enrichment test result in the original paper that showed no common function between three groups of genes. Specifically, genes actively transcribed in the epithelial state were enriched for cell cycle process, while the mesenchymal state was characterized by genes related to cell adhesion. Meanwhile, the partial-EMT state was marked by genes associated with cell motility. Given the results of the GO enrichment test, it is understandable that TRACS assigned the *ECM-receptor interaction pathway* (hsa04512) as the only common function between the partial-EMT and mesenchymal clusters because the interaction between cells undergoing EMT and extracellular matrix (ECM) protein is known to regulate EMT processes including cell adhesion and migration^34^. For example, certain ECM proteins, such as type I collagen, are known to facilitate the EMT process through integrin signalling and disrupt cell-cell adhesions. Furthermore, mesenchymal-like cells migrate along the type I collagen matrix^35^.

In summary, TRACS generated three most distinct clusters that represent epithelial, partial-EMT and mesenchymal states based on the gene expression data with eight time points. Currently, a different number of partial-EMT states in various cancer cell lines have been characterized^36–38^. These studies indicate there would be multiple intermediate states during the EMT process, forming a continuum of cell states. Given the high resolution data with a larger number of (pseudo-)time points such as single cell expression data from EMT, TRACS is expected to help reveal more concrete dynamics of EMT process with multiple partical-EMT states.

### Performance evaluation and comparison with existing tools

The performance of TRACS is evaluated differently in scRNA-seq data and two bulk RNA-seq data. As the scRNA-seq dataset does not provide cluster labels of every gene, the accuracy of clustering cannot be measured with evaluation scores such as F1 score. Therefore, the result was compared with biclustering algorithms to visually inspect the gene expression patterns of clusters. Biclustering is a method to clustering rows and columns of a data matrix simultaneously. With time series gene expression data, biclustering algorithms can be used to generate a gene set related to a certain cluster of time points that might represent a potential cellular state.

Cell cycle and EMT dataset that provide true cluster labels of genes are evaluated with the accuracy of clustering measured by the inferred number of clusters and evaluation scores (F1 score, adjusted Rand index, silhouette score). To see how accurately the Gaussian gap statistics using Gaussian process likelihood can infer the number of clusters, the optimal number of clusters inferred by TRACS is compared with that from the original gap statistics using Euclidean distance. The clustering performance of TRACS is compared with the performance of six algorithms for time series clustering (Supplementary Table S2). Two classic clustering algorithms (K-means and agglomerative clustering) that do not consider time dependency are used in TRACS to verify the independent effect of Gaussian gap statistics on time series. BHC, DPGP and STEM provide the optimal number of clusters with which the evaluation scores are calculated. True $k_{opt}$s are given to K-Shape, GPclust and ClusterNet that take the number of clusters as an input.

#### Single-cell RNA-seq dataset

**Figure 5:**
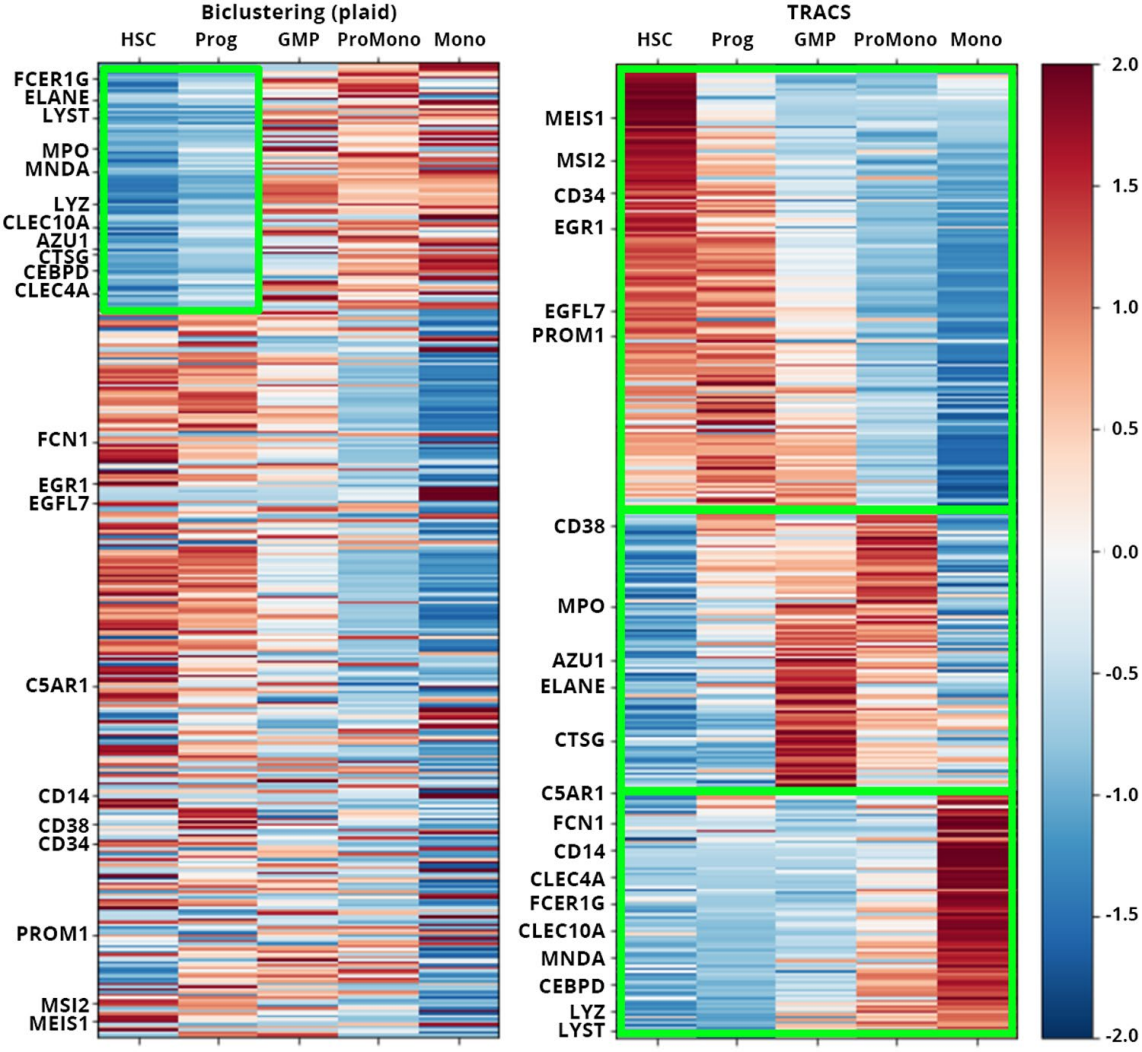
Heatmap of the clustering results of scRNA-seq data by plaid model biclustering algorithm (left) and TRACS (right). Detected clusters are marked as green boxes. 21 cell-type-specific genes that represent successive stages of hematopoietic development are specified on the left side of the heatmaps.

Gene expression generated from scRNA-seq data was analysed with biclustering algorithms implemented in the R package biclust including a plaid model^39^, bimax biclustering^40^, CC biclustering^41^, questmotif biclustering^42^. biclust package was selected as the implemented functions does not require the number of clusters, comparable to TRACS. Figure 5 shows the result of the plaid model biclustering algorithm and TRACS. The plaid model generated one cluster with 91 genes and 2 time points and TRACS detected three clusters, each of which are marked with a green box. Three clusters of TRACS can represent three successive stages of normal hematopoietic development (HSC/Prog, GMP and differentiated myeloid) and 20 out of 21 cell-type-specific genes belong to the correct stages they represent. The cluster detected by the plaid model biclustering included 11 out of 14 cell-type-specific genes of the second and third stages highlighting the inactivated time points of the genes, which shows the algorithm could not detect the individual clusters associated with cell states. The other algorithms generated no cluster (questmotif), one cluster with all genes (CC), or highly overlapping 17 clusters that cannot be visualized (bimax).

#### Cell cycle dataset

**Figure 6:**
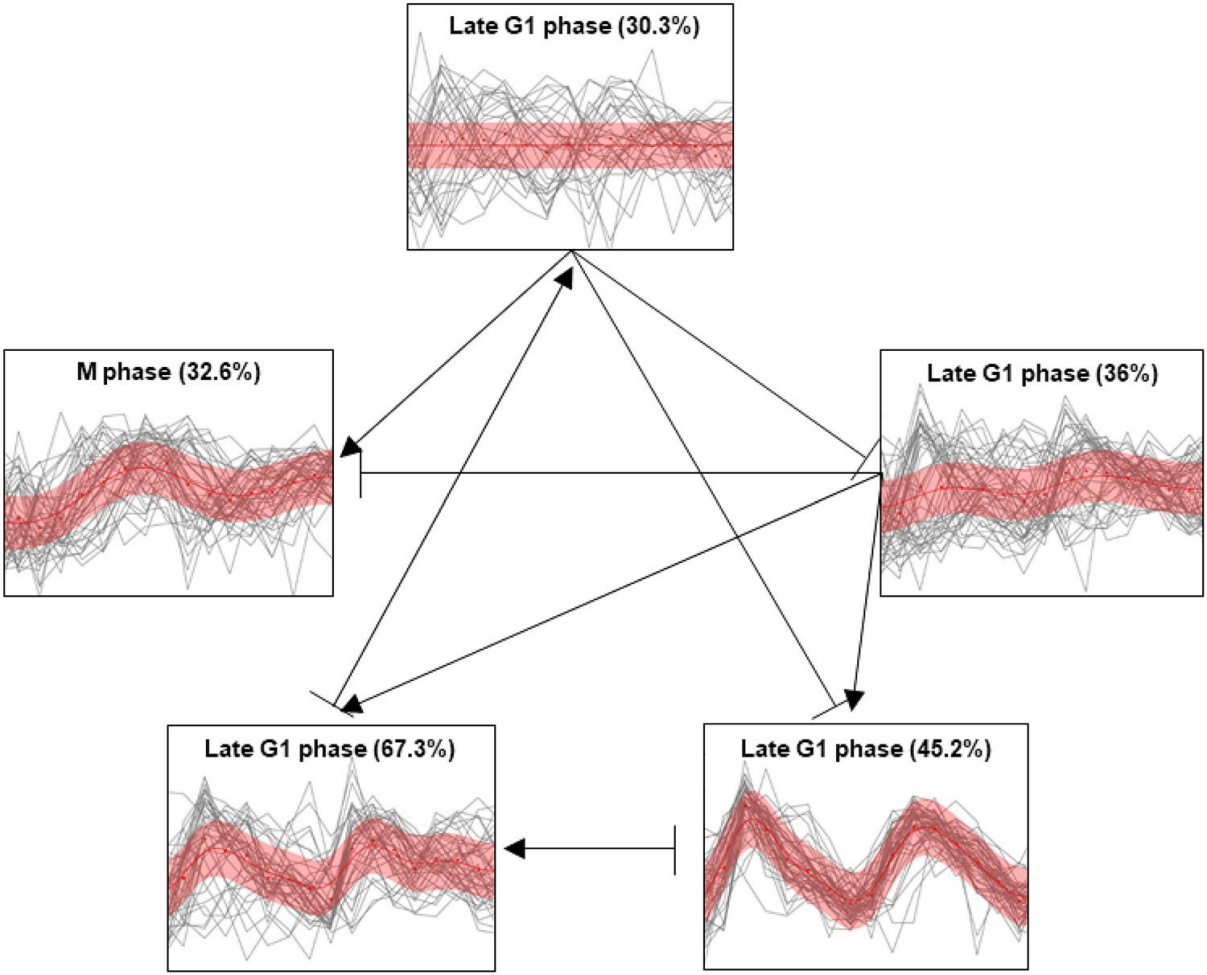
Cluster network of cell cycle dataset inferred by ClusterNet^19^. Triangle arrows and diamond arrows indicate activation and inhibition, respectively. Self-regulations are omitted. Each cluster is labeled with corresponding cell cycle phase based on the true cluster labels of genes. The percentages of genes belonging to the labeled cell cycle phase are marked for each cluster.

Table 1 shows the clustering performance evaluation results with the cell cycle dataset. Gaussian gap statistics using the Gaussian process predicted the number of clusters more accurately (7) than the Euclidean distance (17–18) when the true $k_{opt}$ was 5. Consequently, performance evaluation scores (F1 score, adjusted Rand index, silhouette score) were higher when the $k_{opt}$s from TRACS were closer to 5. BHC and DPGP generated a large number of clusters as 40 and 48, achieving the lowest performance. STEM did not report any significant clusters. The probable reason is that STEM could not distinguish gene distribution in clusters from uniform (random) distribution owing to the large number of possible profiles generated from 17 time points. The K-shape showed the best performance, with true $k_{opt}$ most similar to the TRACS results with 7 clusters. Even though the true $k_{opt}$ was given, the performance scores of GPclust and ClusterNet were lower than those of TRACS. Especially, the clusters produced by ClusterNet show high within-cluster variances, as shown in Table 1 and Fig. 6. In particular, the top cluster in Fig. 6 has no specific expression pattern shared by cluster genes, thereby resulting in a straight line as a mean after Gaussian process regression.

**Table 1:** Optimal number of clusters ($k_{pred}$) inferred from cell cycle dataset ($k_{true}$=5) with a given range of [1, 20] and clustering performance evaluation with inferred $k_{pred}$.

Algorithm	Gap statistics using GP				Gap statistics using ED			
	$k_{pred}$	F1	ARI	Sil.	$k_{pred}$	F1	ARI	Sil.
TRACS (KM)	7	0.536	0.410	0.216	18	0.239	0.158	0.100
TRACS (AC)	7	0.559	0.428	0.218	17	0.353	0.260	0.130
BHC	40	0.119	0.065	− 0.156				
DPGP	48	0.209	0.148	0.087				
STEM	–	–	–	–				

Algorithm	$k_{given}$	F1	ARI	Sil.				
K-shape	5	0.578	0.442	0.261				
GPclust	5	0.410	0.236	0.123				
ClusterNet	5	0.280	0.081	− 0.021				

As K-shape, GPclust and ClusterNet do not predict the number of clusters, the true number of clusters is given ($k_{given} = k_{true}$). (*F1* F1 score, *ARI* adjusted Rand index, *Sil.* Silhouette score, *KM* K-means clustering, *AC* agglomerative clustering, *KS* K-shape).

Using the same gene expression data, a cluster network was generated by ClusterNet^19^ with $k=5$ for comparison with TRACS (Fig. 6). ClusterNet performs clustering and predicts activation ($\rightarrow$) and inhibition ($\dashv$) relationships between clusters. If ClusterNet predicts an activation (inhibition) edge from cluster A to cluster B, this implies that the gene expression profile of cluster B at time point *t* is based on the profile of cluster A at time point $t-1$ multiplied by a positive (negative) weight. As shown in Fig. 6, some of the clusters show high within-cluster variances in gene expression patterns, making it hard to match the clusters with cell cycle phases (Table 1). ClusterNet inferred 10 relationships among 5 clusters, but the relationship is complicated to explain in the absence of a specific order of the clusters.

#### Epithelial-to-mesenchymal transition dataset

The clustering results of the EMT dataset are summarized in Table 2. For TRACS, Gaussian gap statistics using the Gaussian process predicts a more accurate number of clusters (3–4) than Euclidean distance (10), achieving higher values for the F1 score, adjusted Rand index and silhouette score. BHC and DPGP inferred a large number of clusters (624 and 37, respectively) for the EMT dataset, most of which were clusters with a single gene. STEM reported 7 significant clusters out of 50 clusters, which is a default parameter for the maximum number of model profiles. All possible profiles of the EMT dataset are actually $3^{(8-1)}-1=2186$, and 50 out of 2186 clusters are considered candidate profiles. K-Shape showed similar but slightly lower performance than K-means with the same *k*. This suggests that the concept of clustering shifted patterns in K-Shape has less importance in the EMT dataset. STEM selected 7 clusters as significant out of 50 clusters. We evaluated STEM results with true label of 1,386 genes that belong to 7 significant clusters. ClusterNet did not produce clustering results due to an ‘Out of memory’ error, which might stem from the larger number of genes than in the cell cycle dataset.

**Table 2:** Optimal number of clusters ($k_{pred}$) inferred from EMT dataset ($k_{true}$=3) with a given range of [1, 20] and clustering performance evaluation with inferred $k_{pred}$. Abbreviations are same as in Table 1.

Algorithm	Gap statistics using GP				Gap statistics using ED			
	$k_{pred}$	F1	ARI	Sil.	$k_{pred}$	F1	ARI	Sil.
TRACS (KM)	3	0.884	0.796	0.476	10	0.407	0.269	0.233
TRACS (AC)	4	0.768	0.635	0.327	10	0.401	0.258	0.187
BHC	624	0.014	0.007	− 0.373				
DPGP	37	0.152	0.087	− 0.010				
STEM	7	0.470	0.270	0.044				

Algorithm	$k_{given}$	F1	ARI	Sil.				
K-shape	3	0.802	0.632	0.456				
GPclust	3	0.876	0.781	0.478				
ClusterNet	3	–	–	–				

## Conclusions

In this study, TRACS, a novel time series transcriptome data analysis framework, was presented. The novelty of this framework is its ability to generate cell states and transitions of cell states by analysing time series transcriptome data only. TRACS discovers hidden patterns that correspond to hidden cellular states by clustering gene expression data, without any prior knowledge on cell types, cell-type-specific markers or the number of cell states. The proposed algorithm infers cell states with high accuracy by Gaussian process regression, shape-based distance and ranked pairs algorithm. Gaussian gap statistics are implemented in TRACS to infer the optimal number of clusters. The Gaussian gap statistics using the Gaussian process are able to consider time-to-time dependency, leading to the more accurate prediction of the number of clusters. TRACS provides a network of clusters where relationships between clusters are inferred by the shape-based distance, the ranked pairs algorithm and the two-group enrichment test on biological pathways. In three gene expression datasets from scRNA-seq and bulk RNA-seq, TRACS generated correctly ordered networks of clusters, each cluster matching one of the known cell states. Furthermore, several biological studies support the predicted functional link at each cell state change between clusters, showing the utility of TRACS for biological interpretation. Our proposed model is based on the Gaussian assumption for gene expression data that include signal intensities from microarray or log-transformed normalized read counts from RNA-seq. Future work of this study will involve accounting for different types of gene expression data such as raw read counts of RNA-seq that are assumed to follow the negative binomial distribution.

## Methods

### Data pre-processing for feature extraction

TRACS provides an optional step for feature extraction by principal component analysis (PCA) before clustering. PCA^43^ finds the eigenvectors of a covariance matrix of data to project an initial set of features from a high-dimensional space into a reduced set of features while preserving as much of the variation of data as possible. To perform feature extraction on time series, TRACS incorporates the functional PCA^44^ that investigates the modes of variation of functional data and represents the data using a fixed number of eigenfunction basis.

### Gap statistics using distance measure for the Gaussian process for predicting the number of clusters

Gap statistics are used to estimate the optimal number of clusters $k_{opt}$ in a set of data^45^. The algorithm defines ‘gap’ as a difference of within-cluster variance calculated from observed data and from a reference null distribution. An optimal number of clusters $k_{opt}$ in a given range $[1, N_k]$ is determined where the gap between two within-cluster variances is maximized. Different criteria to determine the $k_{opt}$ using gap statistics have been developed such as choosing the minimum *k* where the gap is within the standard deviation of the global maximum gap^46^. Assume that gene expression of one gene is represented as a vector ${\mathbf {x}}=\left\{ x_{iq} | i = 1, \dots , N_t, q = 1, \dots , N_r \right\}$ where $N_t$ is the number of time point and $N_r$ is the number of replicates in each time point. The within-cluster variance $W_k$ given *k* clusters is defined as a sum of squared Euclidean distances between all pairs of expression vectors from each cluster *r* in [1, *k*], which can be calculated by a sum of distances between all data points ${\mathbf {x_r}}$ in the *r*th cluster and as cluster mean ${\mathbf {\mu _r}}$ multiplied by $2n_r$ where $n_r$ is the number of data points in cluster *r* (Eqs. 1, 2). To calculate the distance between data points, averaged values of the replicates at each time point are used such as ${\mathbf {x}}=\left\{ \bar{x_{i}} | i = 1, \dots , N_t \right\}$.

1 $$\begin{aligned} D_r= & {} \sum _{\mathbf {x_i} \in C_r} \sum _{\mathbf {x_j} \in C_r} {|| {\mathbf {x_i}} - {\mathbf {x_j}} ||}^{2} = 2 n_r \sum _{\mathbf {x_r} \in C_r} {|| {\mathbf {x_r}} - {\mathbf {\mu _r}} ||}^{2} \end{aligned}$$

2 $$\begin{aligned} W_k= & {} \sum _{r=1}^{k} \frac{1}{2 n_r} D_r \end{aligned}$$

Gap statistics are calculated as $Gap(k) = E^{*} \left\{ log\left( W_k\right) \right\} - log\left( W_k\right)$ by comparing $log(W_k)$ with $E^{*} \left\{ log\left( W_k\right) \right\}$ that is an expected $log(W_k)$ under a null reference distribution from randomly sampled data. Reference data are sampled within the same range of observed data such that there is no obvious clustering pattern. TRACS uses adapted gap statistics (hereafter referred to as *Gaussian gap statistics*) as a metric for within-cluster variance to reflect temporal dependency in gene expression. We assume that gene expressions in a cluster are generated from a Gaussian process. Cluster mean ${\hat{\mathbf {\mu }}} = \left\{ \hat{\mu _{t_i}}, i=1, \dots , N_t \right\}$ and variance ${\hat{\mathbf {\sigma }}} = \left\{ \hat{\sigma _{t_i}}, i=1, \dots , N_t \right\}$ at each time point $t_i$ are estimated using the expression vectors of all genes ${\mathbf {x}}$ in the cluster including the replicated values. Unlike the original gap statistics that calculates Euclidean distance between a data point and a cluster mean, TRACS uses the log marginal likelihood of a data point being generated from a Gaussian process with ${\hat{\mathbf {\mu }}}$, ${\hat{\mathbf {\sigma }}}$ and noise variance $\sigma ^N$ at time points $P = \{t_i | i \in {1, \dots , N_t} \}$ with kernel matrix ${\mathbf {K}}$. (Eqs. 3, 4).

3 $$\begin{aligned} L_r&= \sum _{{\textbf{x}_{\textbf{r}}} \in C_r} {log P({\textbf{x}_{\textbf{r}}} | P, {\hat{\boldsymbol{\mu}}}, {\hat{\boldsymbol{\sigma}}^2})} \\ &= \sum_{\textbf{x}_{\textbf{r}} \in C_r} - \frac{1}{2} ({\textbf{x}_{\textbf{r}}} - {\hat{\boldsymbol{\mu}}})^T ({\textbf{K}}+{\sigma ^N}^2I)^{-1} ({\textbf{x}_{\textbf{r}}} -{\hat{\boldsymbol{\mu}}}) \\ &\quad -\frac{1}{2} log|{\textbf{K}}+{\sigma^N}^2I|- \frac{N_t}{2} log2\pi \end{aligned}$$

4 $$\begin{aligned}&W_k^{GP} = \sum _{r=1}^{k} L_r \end{aligned}$$

A larger likelihood indicates smaller within-cluster variance. The $k_{opt}$ is determined where the gap between likelihoods of the reference data and of the observed data is maximized, as shown below.

5 $$\begin{aligned} Gap^{GP}(k)= & {} log\left( W_k^{GP}\right) - E^{*} \left\{ log\left( W_k^{GP}\right) \right\} \end{aligned}$$

6 $$\begin{aligned} k_{opt}= \mathop{\text{argmax}}\limits_k Gap^{GP}(k) \end{aligned}$$

The advantage of using the likelihood based on the Gaussian process model is that the likelihood of an observed data point is calculated according to the variance of the time point as well as the distance from means, unlike the original within-cluster variance based on Euclidean distance that considers only the distance between a data point and a cluster mean. Variances and covariances from the Gaussian process are inferred based on the observed data points and a given kernel function that describes the relationship between time points. Supplementary Fig. S2 shows examples of the increased and decreased likelihood of *k* being increased by one when a cluster is divided into two clusters. When the data points are divided into two smooth Gaussian processes and become closer to the new confidence intervals, the likelihood increases. The likelihood decreases when an existing cluster is not properly divided into two clusters when *k* increases. The Gaussian process of a new top cluster has larger variances from regression because the scatteredness of the data points generated an improbable fluctuation. A change in the variances of a Gaussian process results in a decrease of the likelihood, which might not be captured with the Euclidean distance measure.

Gaussian process regression for each cluster is performed with the Scikit-learn^47^ Python package. The radial-basis function kernel $K_R$ and White kernel $K_W$ are jointly used to reflect covariance and Gaussian noise as in Eq. (7). The hyperparameters of kernel function, including the length scale *l* and noise variance $\sigma ^N$, are optimized during fitting of the GaussianProcessRegressor function by maximizing the log-marginal-likelihood, given the range of the length scale as $[0, t_{N_t}]$. When inferring means and variances of a Gaussian process, unobserved time points as well as observed ones are included to interpolate the data. The interval between time points for interpolation is set to 1, where observed time points are integers.

7 $$\begin{aligned} K(t_i, t_j)&= K_{R}(t_i, t_j) + K_{W}(t_i, t_j) \nonumber \\ K_R(t_i, t_j)&= \exp {\left( \frac{- || \frac{t_i}{l} - \frac{t_j}{l} ||^2}{2} \right) } \nonumber \\ K_W(t_i, t_j)&= {\left\{ \begin{array}{ll} {\sigma ^{N}}^2 &{} {\text {if }} t_i= t_j \\ 0 &{} {\text {otherwise}} \end{array}\right. } \end{aligned}$$

After selecting the optimal clustering results with Gaussian gap statistics, TRACS offers users to filter out non-relevant clusters based on the pathway of interest that user provides. When provided with pathway identifier in Kyoto Encyclopedia of Genes and Genomes (KEGG) database^48^, TRACS performs enrichment test on the cluster genes and filters out clusters that are not enriched with pathway genes based on the given threshold of P-value. The filtering process is helpful when the number of genes used in the analysis is large such as using differentially expressed genes (DEGs) detected from the gene expression analysis that are expected to include genes related to other simultaneously activated biological pathways with the pathway of interest.

### Inference of a cluster network by shape-based distance (SBD) and ranked pairs algorithm

Shape-based distance (SBD) is a distance metric developed for time series considering the shifted pattern of two series^15^. Among all possible shifts $w \in [ 1-m, m-1 ]$ between two sequences with the length *m*, SBD selects an optimal shift where the cross-correlation ($CC_w$) is maximized. When SBD is used as a distance metric in a clustering algorithm, such as K-shape^15^, two shifted time series (e.g. Late G1 and S phase genes in Fig. 3) can be merged into a single cluster. In this study, shifted patterns are divided into different clusters and SBD is used after clustering to order every pair of two clusters *a* and *b* with their sample means ${\mathbf {\mu _a}}$, ${\mathbf {\mu _b}}$:

8 $$\begin{aligned} SBD(a, b) = 1 - \max _{w} \frac{CC_w ({\mathbf {\mu _a}}, {\mathbf {\mu _b}})}{\sqrt{CC_o ({\mathbf {\mu _a}}, {\mathbf {\mu _a}}) \cdot CC_o({\mathbf {\mu _b}}, {\mathbf {\mu _b}})}} \end{aligned}$$

By calculating SBD between every pair of clusters, we can derive cluster-cluster distances and their optimal expression shift *w*. We assume cluster *a* is followed by cluster *b* when $w<0$ where *w* is the optimal delay found in *SBD*(*a*, *b*) and vice versa. The order of each pair of clusters can be used to derive the overall order of clusters by ranked pairs algorithm^49^. The ranked pairs algorithm was developed for an electoral system to create a sorted list of winners from the votes comparing each pair of candidates. Using a sequential order of clusters, we can reduce the number of candidate edges of the cluster network from ${\text {K} \atopwithdelims ()2}$ to K-1 edges, where K is the number of clusters.

### Two-group enrichment test for detecting functional similarity between clusters

Once a cluster network is constructed using SBD, the edges between clusters are annotated in terms of the functional similarity between groups of genes in two clusters. The statistical test is to measure the significance of pathways being assigned to clusters. First, we perform a hypergeometric test on every pair consisting of a cluster and a pathway. The hypergeometric distribution is used to model the behaviour of drawing objects (pathway genes) from a bin (cluster). Assume that *N* is the total number of genes and $N_p$ is the total number of pathway genes. The random variable *X* of the hypergeometric distribution represents the number of pathway genes $C_{ip}$ in $C_i$ cluster genes from the total population (Eq. 9). Pathway information is downloaded from KEGG database.

9 $$\begin{aligned} P(X=C_{ip})=f(C_{ip};N,N_p,C_i)=\frac{\left( {\begin{array}{c}N_p\\ C_{ip}\end{array}}\right) \left( {\begin{array}{c}N-N_p\\ C_i-C_{ip}\end{array}}\right) }{\left( {\begin{array}{c}N\\ C_i\end{array}}\right) } \end{aligned}$$

When $C_{ij}$ is the union of clusters *i* and *j*, We define the edge between two clusters if $P(X \ge C_{ijp})<= 0.05 < min(P(X \ge C_{ip}),P(X \ge C_{jp}))$ and $N_p>10$, which means the union of clusters *i* and *j* is enriched in pathway *p*, and an enrichment p-value becomes lower when two clusters are merged, excluding small pathways with 10 or fewer genes. This indicates the ratio of genes relevant to a pathway (function) becomes more significant when two clusters are merged. Cluster genes that meet the criterion are divided into two clusters due to their different expression patterns but they might have activated a same pathway together through the interaction propagation from the previously activated genes to the others over time.

### Performance evaluation of clustering algorithms

To evaluate and compare the performance of clustering algorithms, three metrics are used: F1 score, adjusted Rand index and silhouette score. F1 score and adjusted Rand index compares the ground truth class assignment *X* and the assignment *Y* from the clustering algorithm. The number of pairs of data points that are in the same cluster in *X* and in the same cluster in *Y* is defined as true positive (TP) and the number of pairs of data points that are in the different cluster in *X* and in the same cluster in *Y* is defined as false positive (FP). In a similar fashion, we can define true negative (TN) and false negative (FN) as well. F1 score^50^ is the harmonic mean of *precision* and *recall*:

10 $$\begin{aligned} F1 = 2 \frac{precision \cdot recall}{precision + recall}\quad precision = \frac{TP}{TP + FP}\quad recall = \frac{TP}{TP + FN} \end{aligned}$$

Rand index (RI)^51^ measures the percentage of correct cluster assignments made by the algorithm. However, RI does not guarantee that random assignments have a RI score close to zero. Adjusted Rand index (ARI) is the corrected-for-chance version of RI that establishes a baseline using the expected RI, *E*[*RI*], by a random model.

11 $$\begin{aligned} RI = \frac{TP+TN}{TP+FP+FN+TN}\quad ARI = \frac{RI-E[RI]}{1.0-E[RI]} \end{aligned}$$

Silhouette score^52^ is used to evaluate the clustering results when the ground truth labels are not known. The silhouette coefficient *s*(*i*) for a single data point *i* is defined as:

12 $$\begin{aligned} s(i) = \frac{b(i)-a(i)}{max(a(i),b(i))} \end{aligned}$$

where *a*(*i*) is the mean distance between *i* and all other data points in the same cluster and *b*(*i*) is the smallest mean distance of *i* to all points in any other cluster where *i* is not a member. The silhouette score is the mean *s*(*i*) over all data points, which is higher when clusters are dense and well separated.

## Supplementary Information

Supplementary Information.
