# Supplementary material for: Inferring transcriptomic cell states and transitions only from time series transcriptome data
Source: Sci Rep. 2021 Jun 15;11:12566. doi: 10.1038/s41598-021-91752-9 (PMC8206345; doi:10.1038/s41598-021-91752-9)
Supplement: Supplementary file 1 — Supplementary Information. [file 41598_2021_91752_MOESM1_ESM.pdf]

Supplementary Information for: Inferring transcriptomic cell states and transitions only from time series transcriptome data

Kyuri Jo, Inyoung Sung, Dohoon Lee, Hyuksoon Jang, Sun Kim

1 Supplementary tables

Table S1: The number of genes used (N_g: the number of genes, N_{lg}: the number of labeled genes among N_g) in the cell cycle dataset. The optimal number of clusters (k_{pred}) is predicted by TRACS and the true number of clusters (k_{true}) is compared. The completeness score (S_c) is calculated for genes having a true cell cycle phase label.

Conditions	N_g	N_{lg}	k_{true}	k_{pred}	S_c
Labeled genes	220	220	5	7	0.391
DEGs (P-value <0.01)	750	77	-	11	0.541
DEGs (P-value <0.05)	1,433	137	-	12	0.492

Table S2: Clustering algorithms used to evaluate the accuracy of Gaussian gap statistics.

Algorithm	Designed for TS	Incorporating time points	Automatically detect K	Additional analysis	Ref.
TRACS (proposed)	O	O	O	Cluster network	-
BHC	O	O	O	-	[1]
STEM	O	X	O	GO enrichment test results	[2]
DPGP	O	O	O	-	[3]
K-shape	O	X	X	-	[4]
GPclust	O	O	X	-	[5]
ClusterNet	O	X	X	Cluster network	[6]

2 Supplementary Figures

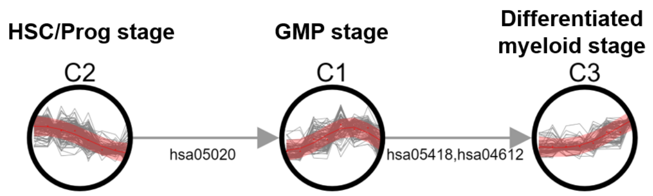

Figure S1: Cluster network of normal BM cells inferred by TRACS. Each cluster is represented with a Gaussian process mean (solid red line) and variance (red area, 95% confidence interval) and edges between clusters are annotated with shared biological pathways.

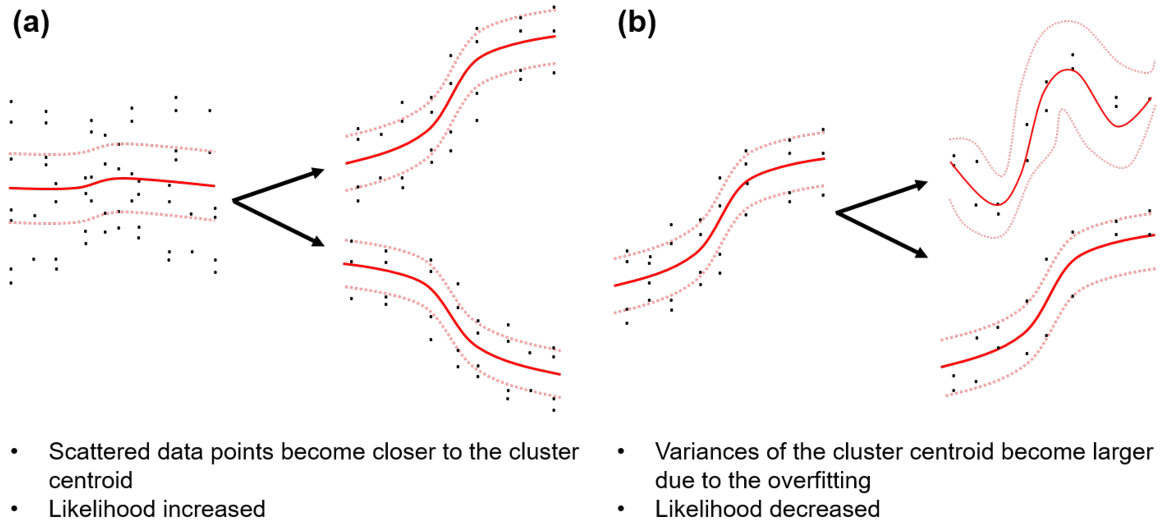

Figure S2: Example of the likelihood (a) increase and (b) decrease. Black dots indicate observed data points. Solid red line and dotted line indicate Gaussian process mean and 95% confidence interval, respectively.

References

[1] Cooke, E. J., Savage, R. S., Kirk, P. D., Darkins, R. & Wild, D. L. Bayesian hierarchical clustering for microarray time series data with replicates and outlier measurements. *BMC bioinformatics* **12**, 399 (2011).

[2] Ernst, J., Nau, G. J. & Bar-Joseph, Z. Clustering short time series gene expression data. *Bioinformatics* **21**, i159–i168 (2005).

[3] McDowell, I. C. *et al.* Clustering gene expression time series data using an infinite gaussian process mixture model. *PLoS computational biology* **14**, e1005896 (2018).

[4] Paparrizos, J. & Gravano, L. k-shape: Efficient and accurate clustering of time series. In *Proceedings of the 2015 ACM SIGMOD International Conference on Management of Data*, 1855–1870 (ACM, 2015).

[5] Hensman, J., Rattray, M. & Lawrence, N. Fast nonparametric clustering of structured time-series. *Pattern Analysis and Machine Intelligence, IEEE Transactions on* **PP** (2014).

[6] Shiraishi, Y., Kimura, S. & Okada, M. Inferring cluster-based networks from differently stimulated multiple time-course gene expression data. *Bioinformatics* **26**, 1073–1081 (2010).
